# Diagnostic value of exfoliated tumor cells combined with DNA methylation in bronchoalveolar lavage fluid for lung cancer

**DOI:** 10.1097/MD.0000000000034955

**Published:** 2023-09-08

**Authors:** Huiling Lu, Dang Lin

**Affiliations:** a Department of Pulmonary and Critical Care Medicine, Affiliated Suzhou Municipal Hospital of Nanjing Medical University, Jiangsu, China.

**Keywords:** diagnosis, DNA methylation, exfoliated tumor cells (ETCs), lung cancer, RASSF1A, SHOX2

## Abstract

**Background:**

To evaluate the diagnostic value of exfoliated tumor cells (ETCs) numbers combined with DNA methylation levels in bronchoalveolar lavage fluid (BALF) in lung cancer.

**Methods:**

BALF samples were collected from 43 patients with lung cancer and 23 with benign lung disease. ETCs were detected by the nano-enrichment method, and the methylation status of the short stature homeobox gene 2 (SHOX2) and the RAS association domain family 1, isoform A (RASSF1A) gene were detected by RT-PCR. The diagnostic value of each metric was evaluated by receiver operating characteristic curve analysis, specificity and sensitivity.

**Results:**

The sensitivity/specificity of RASSF1A and SHOX2 methylation detection were 44.12%/76.47% and 93.75%/87.50%, respectively. When “RASSF1A/SHOX2 methylation” was used as a positive result, the sensitivity increased to 88.24%, and the specificity decreased to 81.25%. When “RASSF1A + SHOX methylation” was used as positive, the sensitivity was reduced to 32.35%, but the specificity was increased to 100.00%. The sensitivity and specificity of ETCs detection in BALF were 89.47% and 16.67%, respectively. When “SHOX2/RASSF1A methylation + ETCs was used as a positive result, the sensitivity and specificity of the detection were 79.31% and 81.82%, respectively. When “SHOX2 + RASSF1A + ETCs” was used as positive, the sensitivity was 34.48% and the specificity was 90.91%. Receiver operating characteristic curve analysis showed that when SHOX2, RASSF1A methylation and ETCs were combined, the diagnostic sensitivity increased to 0.778.

**Conclusion:**

ETCs counting in combination with SHOX2 and RASSF1A methylation assays in BALF samples has demonstrated excellent sensitivity for lung cancer diagnosis and is an effective complementary tool for clinical diagnosis of lung cancer.

## 1. Introduction

Lung cancer is a malignant tumor that seriously endangers human health and life, with a 5-year relative survival rate of only 19%.^[1]^ Early diagnosis and appropriate treatment are effective and necessary to prolong the life of patients with lung cancer. Low-dose computed tomography is the main modality for clinical detection of lung cancer, but its high false-positive rate leads to an unnecessary treatment burden.^[2]^ Compared with traditional blood, bronchoalveolar lavage fluid (BALF) has become an alternative source of lung cancer biomarkers because of its proximity to tumor cells.^[3]^ In addition, BALF can be obtained using minimally invasive methods, making it potentially useful for clinical applications in lung cancer diagnosis.^[4,5]^

DNA methylation is an epigenetic modification critical for human development and disease.^[6]^ What even, DNA methylation is also involved in tumor formation during the early stages of tumorigenesis.^[7]^ With the development of highly sensitive DNA methylation detection technologies, aberrant methylation status has become an attractive biomarker for cancer diagnosis.^[8]^ Detection of the methylation patterns of the short stature homeobox gene 2 (SHOX2) and the RAS association domain family 1, isoform A (RASSF1A) has been used in the diagnosis of lung cancer.^[9]^ Hypermethylation of the SHOX2 and RASSF1A in plasma is highly correlated with the diagnosis and prognosis of lung cancer; however, the combined detection of SHOX2 and RASSF1A in BALF has rarely been reported.^[10]^

Isolation and detection of exfoliated tumor cells (ETCs) from BALF, has been proved to be a valuable way in the diagnosis of lung cancer.^[11]^ However, the sensitivity of the commonly used cytological examination methods is low, which reduces the application value of BALF in the diagnosis of lung cancer.^[12]^ Therefore, the key to improve the application of BALF liquid biopsy in the clinical diagnosis of lung cancer is to improve the separation efficiency of ETCs.^[13]^ In recent years, counting and characterization of CTC has attracted great interest from the scientific community,^[12–14]^ and has been shown to be independently predictive of lung cancers.^[15]^ Previous studies have confirmed that the diagnostic efficacy of BALF and peripheral blood in differentiating benign lesions from lung cancer is consistent with that of histopathology.^[11,16]^ In this study, the highly efficient method for CTC isolation found in the study by Shen et al, was used to isolate ETCs from BALF samples.^[17,18]^ To make up for its low sensitivity and improve the application of ETCs as more effective biomarkers for early tumor diagnosis.

DNA methylation detection and ETCs detection alone may not provide sufficient diagnostic accuracy for lung cancer. This study aimed to investigate whether DNA methylation biomarker detection combined with ETCs detection in BALF samples can more effectively identify lung cancer and controls (normal individuals, benign lung disease, and patients with alternative cancer).

## 2. Material and methods

### 2.1. Patients and BALF preparation

In this study, we recruited 66 patients who underwent fiber-optic bronchoscopy examination at the Affiliated Suzhou Hospital of Nanjing Medical University from December 2018 to June 2019. Sample information was recorded on the sample collection forms. BALF samples were collected by washing the affected lung segment with 20 to 40 mL of normal saline during fiber-optic bronchoscopy examinations. None of the subjects received chemotherapy, radiotherapy, or surgical intervention before BALF collection. Informed consent was obtained from all participants, and all protocols regarding the use of patient samples were approved by the hospital ethics review committee. The patients received treatment in accordance with the Declaration of Helsinki. The final diagnosis of all patients was made based on historical results.

### 2.2. DNA extraction and methylation detection

A preparation of 10 mL BALF samples centrifuged at 10,000 rpm for 5 minutes was used for genomic DNA extraction. The genomic DNA was treated with bisulfite, which converts unmethylated cytosines into uracil, whereas methylated cytosines remained unchanged during treatment. The Applied Biosystems 7500 Sequence Detection System was used for quantitative real-time PCR. DNA extraction and methylation detection were performed using the Methylated Human SHOX2 and RASSF1A Gene Detection Kit (Tellgen Co., Ltd., Shanghai, China) which was used to detect the methylation levels of CpG islands in the SHOX2 and RASSF1A promoter regions. Methylated SHOX2 and RASSF1A DNA plasmids were used as the controls.^[10]^

The quantitative real-time PCR results were interpreted according to the manufacturer’s instructions. An amplification curve of the FAM fluorescence signal with a smooth “S” shape and a threshold cycle (CT) < 35 indicated a positive result for RASSF1A methylation; CT ≥ 35 indicated a negative result for RASSF1A methylation. An amplification curve of the VIC fluorescence signal with a smooth “S” shape and a CT < 32 indicated a positive result for SHOX2 methylation; a CT ≥ 32 indicated a negative result for SHOX2 methylation. “/” indicates that a positive RASSF1A or SHOX2 methylation; “+” indicates that both the RASSF1A and SHOX2 methylation.

### 2.3. ETCs capture and detection

Approximately 7.5 mL of BALF samples for ETCs capture, which was performed according to Wei et al.^[17]^ BALF was gently mixed with 1 × RBC lysis buffer at 1:10 and incubated at room temperature for 15 minutes. The mixture was then centrifuged at 200 g for 5 minutes and the supernatant was discarded. The precipitate was resuspended in 2 mL of 1% FPBS and repeated once. Transferred all resuspensions (cells) to the culture dish and cultured at 37˚C for 1 hour, then changed temperature to 4˚C for another 10 minutes. Subsequently, replaced the medium with 4% paraformaldehyde and cultured for 10 minutes at 4˚C. Lastly, replaced 4% paraformaldehyde with precooled methanol and cultured at −20˚C for 10 minutes. The methanol was removed and the substrate was rinsed with PBS 3 times. An appropriate amount of 2% skimmed milk in PBS was added to the substrate for blocking, followed by the addition of anti-pancytokeratin-cy5, anti-CD45-FITC antibodies for incubation overnight at 4˚C (both antibody concentrations were prepared with the skimmed milk, which described above). On the second day, the dish was cleaned 3 times with PBS and the nuclei were labeled with DAPI.

Images of ETCs were captured using a Cytell cell imaging system. The treated samples were scanned by the high-throughput capture Cytell Cell Imaging System (GE Healthcare Life Sciences). Five hundred random scan points for each sample were captured using 3 different emission spectra.^[19]^ DAPI^+^, CK^+^ and CD45^−^ cells were identified as ETCs. The number of ETCs was counted using the supporting software.

### 2.4. Statistical analysis

The diagnostic value of the combined method was evaluated with sensitivity and specificity. Sensitivity is defined as the ratio of correctly assigned positive lung cancer samples to all the lung cancer samples. Specificity is defined as the proportion of correctly allocated negative samples in all normal/benign lung and other cancer samples. The sensitivity and specificity of methylation and ETCs detection of BLAF samples were calculated according to the study of Emma S. et al.^[20]^ Receiver operating characteristic (ROC) and area under the ROC curve (AUC) were analyzed using the IBM SPSS Statistics 27 (Armonk, NY) and MedCalc software, and the logistic regression fitting formula was used to calculate the predicted probability of the combined scheme. Statistical significance was defined as *P* < .05.

## 3. Results

### 3.1. Clinicopathologic data

The clinicopathologic data of these 66 patients were summarized in Table 1, including gender, age, histological subtype, and tumor stage.

**Table 1:** Demographic and clinical features of the patients.

	Total		Lung cancer		Control	
	n	%	n	%	n	%
Gender	66		43		23	
Male	52	78.8%	35	81.4%	17	73.9%
Female	14	21.2%	8	18.6	6	26.1%
Age (yr)						
≤ 50	8	12.1%	1	2.3%	7	30.4%
51–60	12	18.2%	10	23.3%	2	8.7%
61–70	25	37.9%	19	44.2%	6	26.1%
≥ 71	21	31.8%	13	30.2%	8	34.8%
Median age	63		65		59	
Age range	17–83		46–83		17–83	
Histology subtype						
Squamous cell carcinoma	-		23	53.5%	-	
Adenocarcinoma	-		13	30.2%	-	
Small cell lung cancer	-		4	9.3%	-	
Large cell lung cancer	-		2	4.7%	-	
Unknown	-		1	2.3%	-	
Benign lung diseases	-		-		20	87.0%
Malignancies in other	-		-		3	13.0%
Tumor stage						
Stage I/II	-		11	25.6%	-	
Stage III/IV	-		29	67.4%	-	
Unknown	-		3	7.0%	-	

(1) Benign lung diseases including pulmonary infection, masses and bronchiectasis etc; (2) Malignancies in other systems including thyroid carcinoma and esophageal cancer.

**Figure 1.**
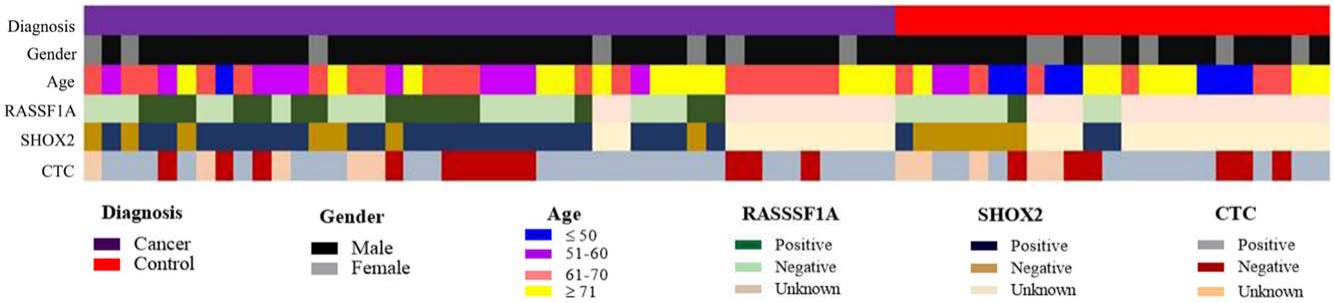
Results of alveolar lavage fluid methylation and ETCs detection. ETCs = exfoliated tumor cells.

The current study included 43 patients with lung cancer and 23 patients with benign lung disease. Benign lung diseases including pulmonary infection, masses and bronchiectasis etc. Briefly, there were 52 (78.8%) males and 14 (21.2%) females with a median age of 63 years (range 17–83 years). Fifty samples were tested for methylation, including 34 lung cancer patients and 16 healthy controls. Among them, 33 samples showed at least 1 methylation of SHOX2 and RASSF1A genes, 17 samples showed SHOX2 methylation, 5 samples showed RASSF1A methylation, and 11 samples showed both SHOX2 and RASSF1A methylation. SHOX2 and RASSF1A genes were not methylated in 17 samples. A total of 56 samples were tested for ETCs, including 38 patients with lung cancer and 18 controls. Among them, 49 samples with the number of ETCs ≥ 5/7.5 mL were recorded as positive, and 7 samples with the number of ETCs < 5/7.5 mL were recorded as negative (Fig. 1).

### 3.2. Diagnostic sensitivity of DNA methylation and ETCs in BALF in different groups

The sensitivity and specificity of methylation and ETCs detection in BLAF samples are shown in Table 2. The sensitivities of RASSF1A and SHOX2 were 44.12% and 76.47%, and the specificities were 93.75% and 87.50%, respectively; indicating that the performance of BLAF in detecting RASSF1A methylation was inferior to that of SHOX2. When any methylation of RASSF1A/SHOX2 was set to be positive, compared with the single result, the sensitivity increased to 88.24%, and the specificity decreased to 81.25%. When RASSF1A + SHOX2 were simultaneously methylated as positive results, the sensitivity decreased to 32.35%, but the specificity reached 100.00% compared to a single result. The sensitivity and specificity of ETCs detection of free tumor cells in BLAF were 89.47% and 16.67% respectively. The sensitivity and specificity of SHOX2/RASSF1A combined with methylation and ETCs positivity were set as positive results, and the sensitivity and specificity of detection were 79.31% and 81.82%, respectively. The sensitivity and specificity of SHOX2/RASSF1A combined with ETCs positivity were set as positive results, with a sensitivity of 34.48% and a specificity of 90.91%.

**Table 2:** The sensitivities and specialties of DNA methylation, ETCs detection and the combined assays on lung cancer patients.

Group	AUC (95% CI)	Sensitivity% (SE)	Specificity% (SP)
RASSF1A	0.679 (0.515–0.816)	46.9 (29.1–65.3)	88.9 (51.8–99.7)
SHOX2	0.724 (0.562–0.852)	78.1 (60.0–90.7)	66. 7 (29.9–92.5)
ETCs	0.532 (0.394 –0.667)	84.2 (68.7–94.0)	22.2 (6.4–47.6)
RASSF1A/SHOX2	0.731 (0.570–0.857)	90.6 (75.0–98.0)	55.6 (21.2–86.3)
RASSF1A + SHOX2	0.672 (0.508–0.810)	34.4 (18.6–53.2)	100.0 (66.4–100.0)
RASSF1A/SHOX2 + ETCs	0.759 (0.579–0.890)	92.6 (75.7–99.1)	50.0 (11.8–88.2)
RASSF1A + SHOX2 + ETCs	0.778 (0.600–0.903)	55.6 (35.3–74.5)	100.0 (54.1–100.0)

AUC = area under the curve, ETCs = exfoliated tumor cells, RASSF1A = the RAS association domain family 1, isoform A, SHOX2 = the short stature homeobox gene 2.

### 3.3. ROC curve analysis of the DNA methylation and ETCs in BALF

**Figure 2.**
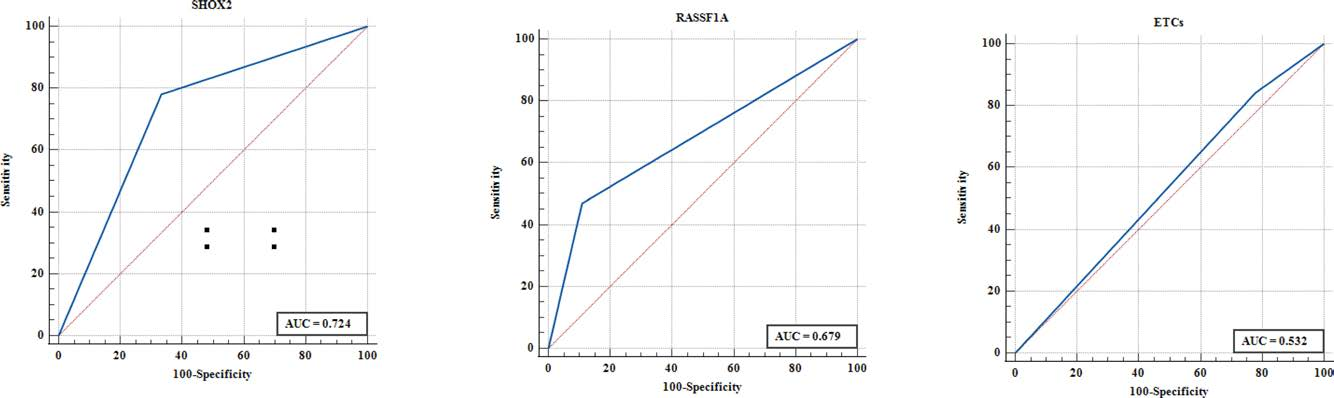
ROC analysis of SHOX2, RASSF1A methylation and ETCs assays on patients. (A) ROC analysis of SHOX2 methylation detection, (B) ROC analysis of RASSF1A methylation detection, and (C) ROC analysis of ETCs detection. ETCs = exfoliated tumor cells, RASSF1A = the RAS association domain family 1, isoform A, ROC = receiver operating characteristic curve, SHOX2 = the short stature homeobox gene 2.

**Figure 3.**
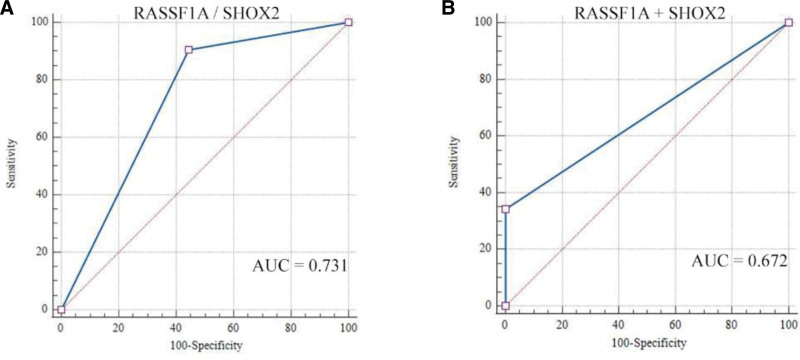
ROC analysis of SHOX2, RASSF1A methylation assays on patients. (A) ROC analysis of SHOX2/ RASSF1A methylation detection, (B) ROC analysis of SHOX2 + RASSF1A methylation detection. RASSF1A = the RAS association domain family 1, isoform A, ROC = receiver operating characteristic curve, SHOX2 = the short stature homeobox gene 2.

**Figure 4.**
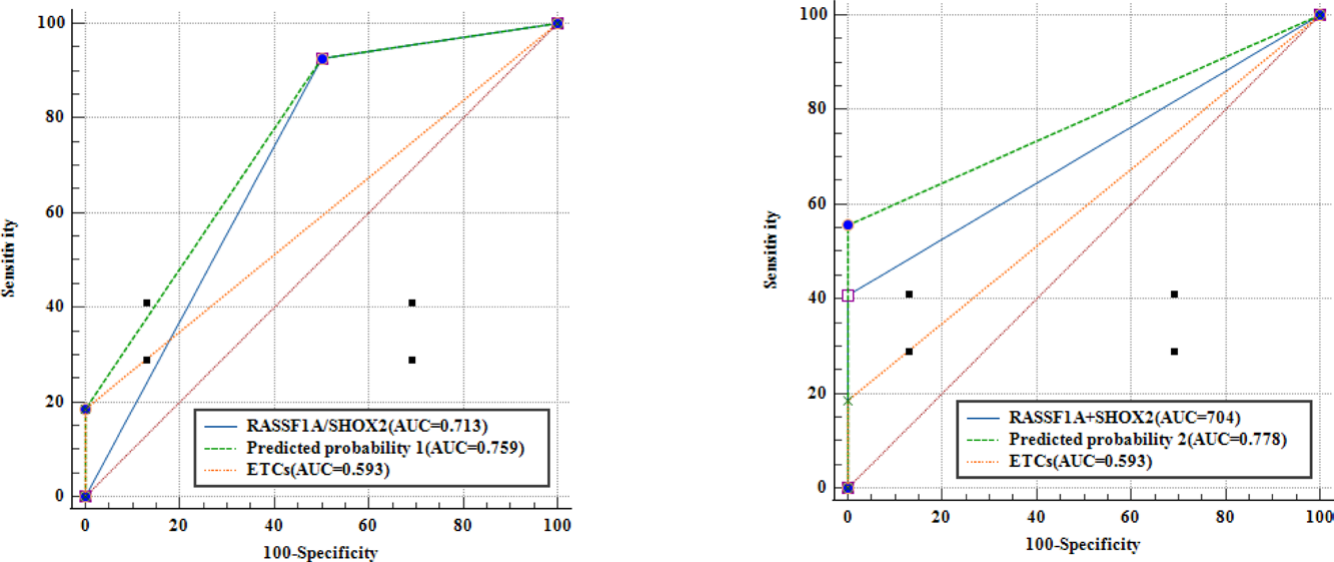
ROC analysis of SHOX2, RASSF1A methylation and ETCs assays on patients. (A) ROC analysis of SHOX2/ RASSF1A methylation combined ETCs detection, (B) ROC analysis of SHOX2 + RASSF1A methylation combined ETCs detection. ETCs = exfoliated tumor cells, RASSF1A = the RAS association domain family 1, isoform A, ROC = receiver operating characteristic curve, SHOX2 = the short stature homeobox gene 2.

ROC curve analysis was performed to compare the diagnostic efficacy of SHOX2, RASSF1A methylation and ETCs detection in BALF samples. As shown in Figure 2, SHOX2 methylation in BALF showed the highest AUC value of 0.724, compared to RASSF1A (AUC value: 0.679) and ETCs (AUC value: 0.532). As shown in Figure 3, the AUC value of SHOX2 or RASSF1A methylation was higher than the AUC value of SHOX2 and RASSF1A methylation in BALF. Notably, when combining SHOX2 or RASSF1A methylation with ETCs, the AUC was 0.759; when combining SHOX2 and RASSF1A methylation with ETCs, the AUC was 0.778 (Fig. 4).

## 4. Discussion

Lung cancer is one of the most common malignant cancers worldwide, and its early diagnosis is very important for the prognosis of patients.^[21]^ At present, the emerging molecular diagnostic methods of lung cancer have attracted wide attention due to their sensitivity and objectivity.^[22]^ Studies have shown that SHOX2 and RASSF1A aberrant methylation detection has an important link with cancer diagnosis.^[23,24]^ Methylation analysis of these 2 genes in plasma samples has been performed extensively, but the sensitivity is unsatisfactory (SHOX2 and RASSF1A sensitivity is 60% and 52.4%, respectively).^[25]^ BALF is a noninvasive specimen that is easily obtained by fiber-optic bronchoscopy and is routinely examined in patients with suspected lung cancer. Owing to its proximity to tumor cells, it has become an important alternative sample type for cancer diagnostic markers. Thus, the methylation analysis of SHOX2 and RASSF1A in BALF samples is of great significance for the diagnosis of lung cancer.^[26]^ Studies have confirmed that the detection and counting for CTCs can be used as an important biomarker for clinical staging, prognosis, and treatment predictions of lung cancer.^[27]^ The number of CTCs may be closely related to the prognosis of patients with lung cancer; the higher the number of CTCs, the shorter the survival time of patients.^[28]^ Research confirms that the diagnostic efficacy of ETCs in BALF and CTC in peripheral blood in differentiating benign lesions from lung cancer is consistent with that of histopathology.^[11]^ Therefore, we hypothesized that the combined detection of ETCs and DNA methylation in BALF may improve the diagnostic sensitivity of lung cancer.

In our study, we compared the diagnostic power of ETCs alone and DNA methylation assays in lung cancer patients. The diagnostic power of CTC for lung cancer was very low (AUC = 0.532), and the diagnostic power of DNA methylation analysis for lung cancer was slightly higher (AUC = 0.672). The combined analysis of ETCs and DNA methylation in BALF (AUC = 0.778) showed the best diagnostic power in lung cancer diagnosis compared with ETCs detection and DNA methylation detection alone. In other words, the assessment of lung cancer cannot be performed using a single ETCs or DNA methylation analysis.^[10,17]^

Sensitivity is the chance of not misdiagnosing (false negative) if a condition is diagnosed. In other words, specificity is the chance of not misdiagnosing (positive) if the index is diagnosed. If an independent index value increases its diagnostic sensitivity, it must reduce its diagnostic specificity, that is to say, reducing misdiagnosis must improve misdiagnosis, and vice versa. The research results suggest that the sensitivity of combined diagnosis was low (55.6%), but the specificity was 100%. From another point of view, improving the probability of no misdiagnosis has certain diagnostic application value. A relevant study revealed that biomarkers in BALF had features such as earlier appearance and higher concentrations than those in serum samples.^[29]^ The sensitivity and specificity of combined with DNA methylation detection were 79.31% and 81.82%, respectively.

Methylation results from BLAF in patients with squamous cell carcinoma, adenocarcinoma, and small cell lung cancer are also summarized and analyzed. There was no difference in positive DNA methylation rates between squamous cell carcinomas and adenocarcinomas. However, there are some limitations to our study. Only 63 patients were included in the study, and more patients need to be enrolled for further study. In addition, the control group should consist of a group of healthy cases to assess specificity and sensitivity, but obtaining BALF samples from healthy individuals is more difficult. Moreover, it is unclear whether the methylation changes in these 2 genes combined with ETCs number in BLAF samples are associated with the prognosis of lung cancer patients.

## 5. Conclusion

ETCs counting in combination with SHOX2 and RASSF1A methylation assays in BALF has shown to be highly efficient in lung cancer diagnosis and is an effective complementary tool for clinical diagnosis of lung cancer.

## Author contributions

**Conceptualization:** Hui ling Lu, Dang Lin.

**Data curation:** Hui ling Lu, Dang Lin.

**Funding acquisition:** Hui ling Lu.

**Investigation:** Hui ling Lu, Dang Lin.

**Methodology:** Hui ling Lu, Dang Lin.

**Validation:** Hui ling Lu, Dang Lin.

**Writing – review & editing:** Dang Lin.
